# A Multidisciplinary Approach for Rehabilitation of Enucleated Sockets: Ocular Implants with Custom Ocular Prosthesis

**DOI:** 10.7759/cureus.2201

**Published:** 2018-02-16

**Authors:** Minati Choudhury, Fathima Banu, Shanmuganathan Natarajan, Anand Kumar, Padmanabhan TV

**Affiliations:** 1 Division of Restorative Dentistry,school of Dentistry, International Medical University; 2 Department of Prosthodontics, Faculty of Dental Sciences, Sri Ramachandra University, Porur, Chennai, India

**Keywords:** ocular implant, custom ocular prosthesis, characterization, special ocular impression

## Abstract

Interdisciplinary prosthodontics goes beyond our imagination into fields that have a direct effect on our total body health and quality of life. Removal of an eye has a detrimental effect on the psychology of the patient. Enucleation involves removal of the eyeball proper and leads to an enophthalmic socket with a shrunken eye, which has a crippling effect on patient’s emotional and social life. Custom-made eye prosthesis simulates the characteristics of the companion eye and helps in restoring the normal facial appearance. Restoration of saccadic eye movements occurring during speech is desirable because this greatly contributes to a normal facial expression. This can be achieved by an orbital implant, which helps in orbital volume replacement and restoration of prosthesis movement and comfort. This article describes prosthodontic rehabilitation of enucleated eye sockets with orbital implants for two patients.

## Introduction

Loss of an eye due to trauma, cancer, or congenital defects creates a deep psychological impact on a person. An artificial eye should be given as soon as possible to help these patients heal medically and emotionally. With advanced medical and dental technology, the modern day artificial eye can be as natural as the lost eye. It has two important components. First is an orbital implant which is placed in the anophthalmic socket at the time of surgical removal of the eye to replace the lost orbital volume and to maintain the remaining extraocular muscle movements to aid in a better performance of the ocular prosthesis. Evisceration, enucleation, and exenteration are the three general surgical treatment procedures used as the primary treatment modality in the surgical removal of the eye [1]. These procedures lead to tissue constriction around the ocular cavity with complications such as enophthalmos and superior sulcus defect [2]. Placement of orbital implants during the surgery reduces the chances of contracture and volume deficit and restores the eye movements. The second component is the ocular prosthesis that fits over the orbital implant and simulates a perfectly healthy companion eye and restores the normal saccadic eye movements during speech and improves facial expression [3-4]. It can be prefabricated or custom made. A custom ocular prosthesis achieves intimate contact with the tissue bed which helps in restoring natural eye movements without pain or discomfort which is a drawback in prefabricated prosthesis [5]. We present two case reports of a custom ocular prosthesis with orbital implants.

## Case presentation

Case 1

**Figure 1:**
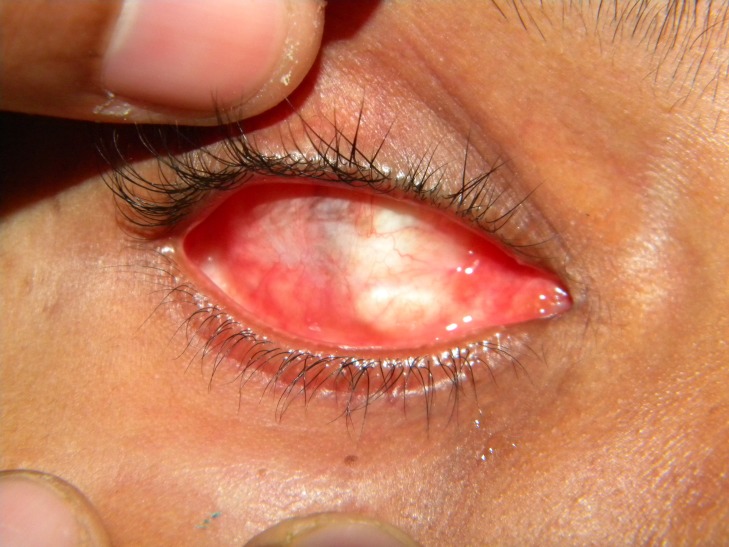
Enucleated ophthalmic socket

A 19-year-old female patient was referred to the Department of Prosthodontics from Ophthalmology for the replacement of missing left eye (Figure 1).

The patient's history revealed a trauma to the left eye at the age of three followed by lens atrophy. An evisceration of the left eye was done followed by placement of an acrylic orbital implant (polymethyl methacrylate implant, 10 mm) (Gulden Ophthalmics, Elkins Park, PA) in the scleral pocket anterior to Tenon’s capsule. The size of the implant depends on the dimension of the orbit. The prefabricated ocular prosthesis was initially inserted. Due to uncoordinated movements and inflammation of the orbital region with increased lacrimation, a custom-made ocular prosthesis was advised.

Case 2

A 24-year-old female patient with traumatic perforating injury to right eye eight years back developed into a large ciliary and intercalary staphyloma, reported to the Department of Ophthalmology three years after the injury. The contents of the eyeball were eviscerated, preserving the sclera and conjunctiva, following which an acrylic orbital implant was placed in the scleral pocket; a prefabricated ocular prosthesis was given six weeks after implant placement. She subsequently developed inflammation in the sclera and was unable to adapt to the prefabricated prosthesis. She was later referred to the Department of Prosthodontics for a custom-made prosthesis.

**Figure 2:**
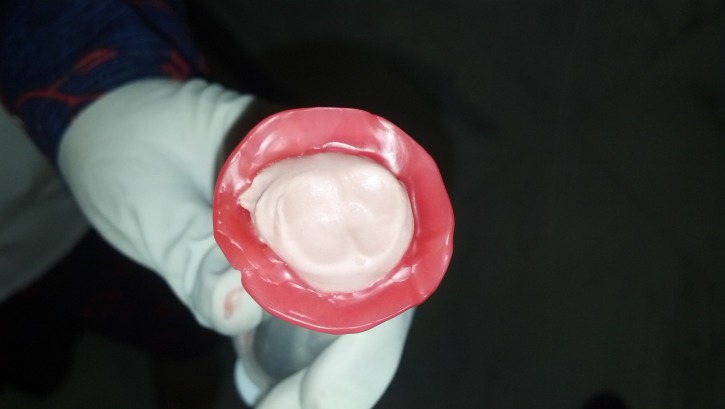
Primary impression with irreversible hydrocolloid

**Figure 3:**
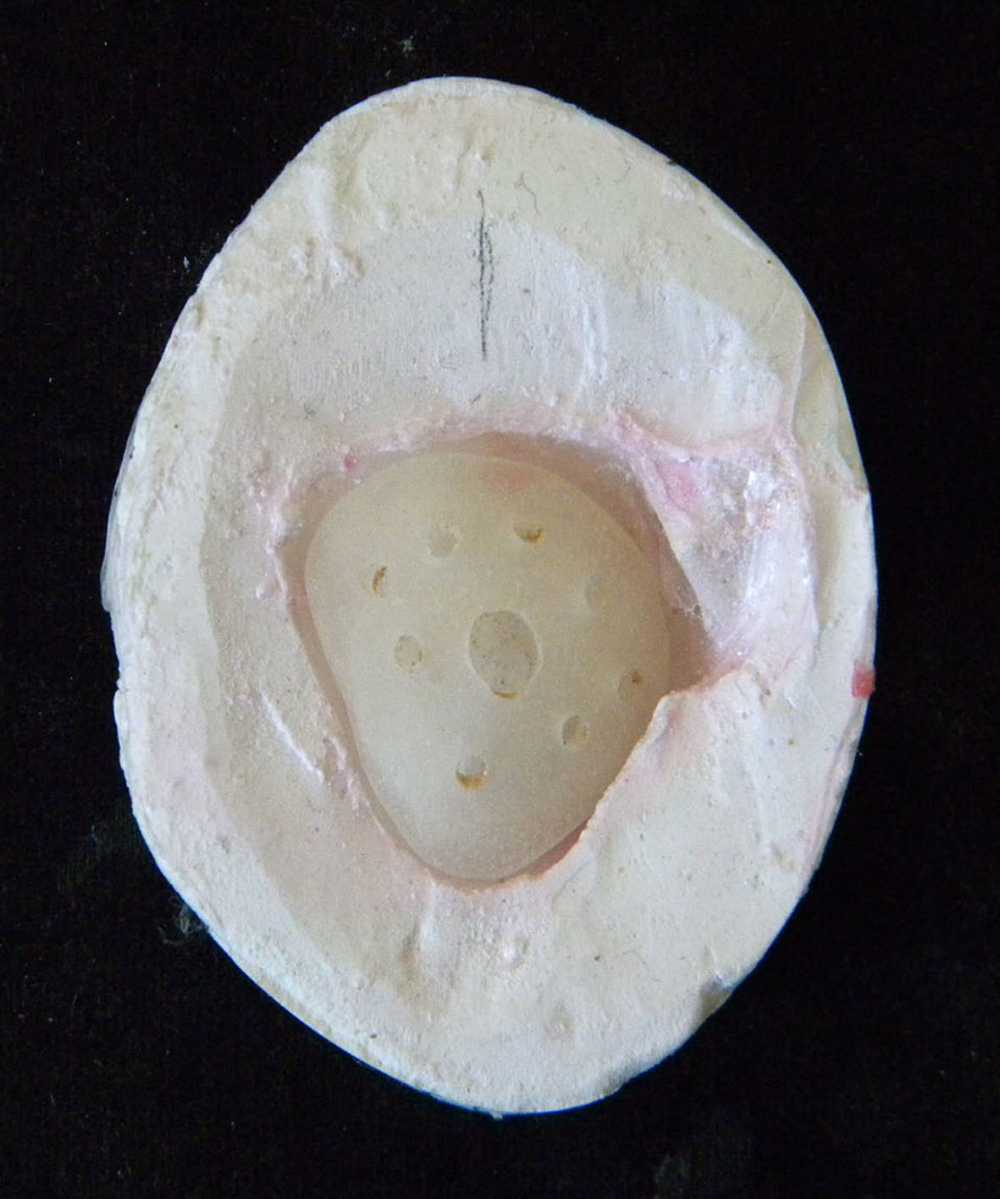
Custom ocular special tray

For prosthesis fabrication, a custom-made prosthesis requires an accurate impression of the defect. Hence, a two-stage impression technique was preferred in both the cases. The preliminary impression was taken with irreversible hydrocolloid (Zelgan) (Zhermack SpA, Italy) (Figure 2), and a self-cure clear acrylic resin special tray was fabricated (DPI-RR Cold Cure) (Dental Products of India, Mumbai) (Figure 3).

**Figure 4:**
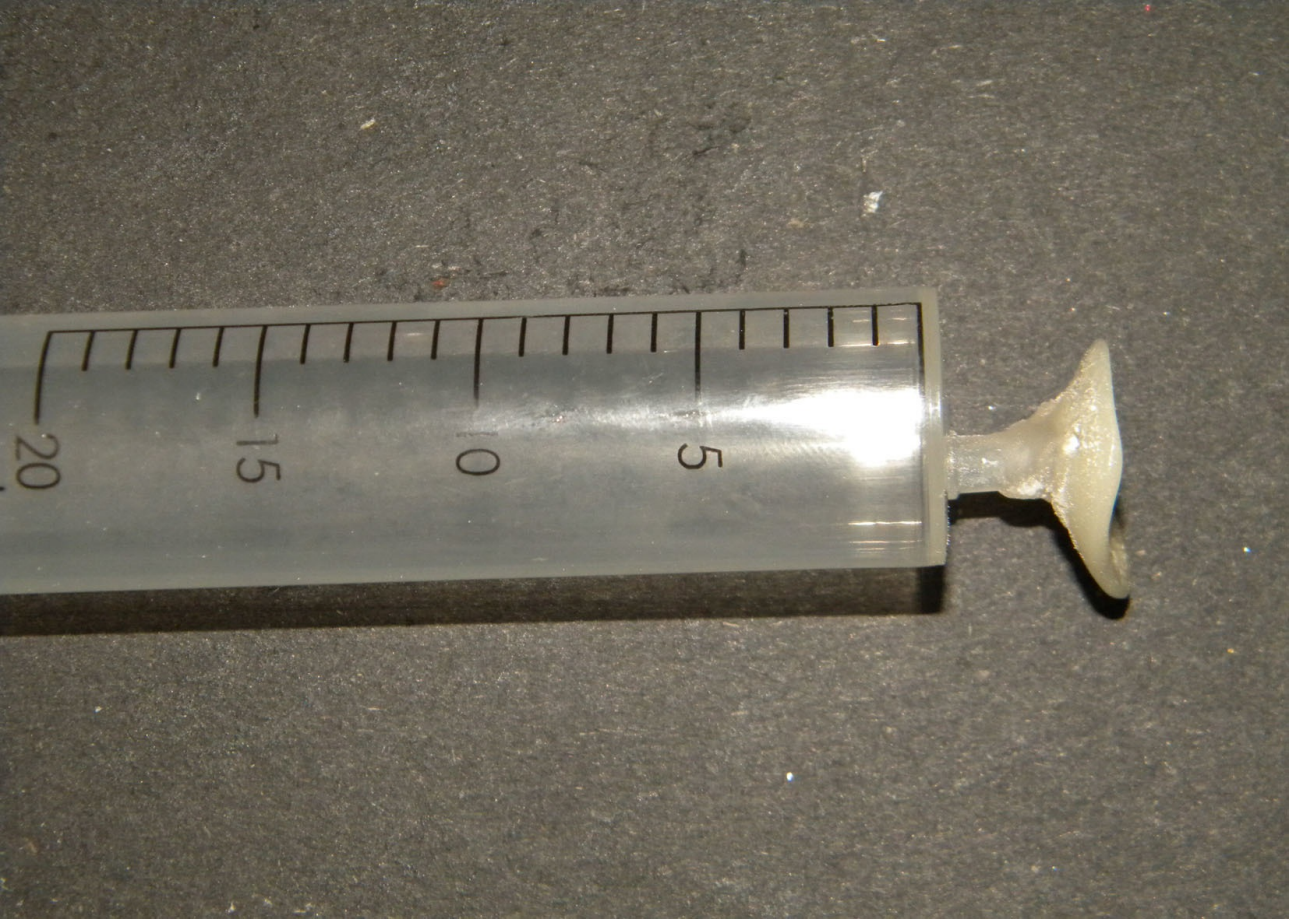
Special tray with syringe for secondary impression

In the area corresponding to the iris, a 10 ml syringe was attached at a right angle and the tray was tried in the socket for accuracy (Figure 4).

**Figure 5:**
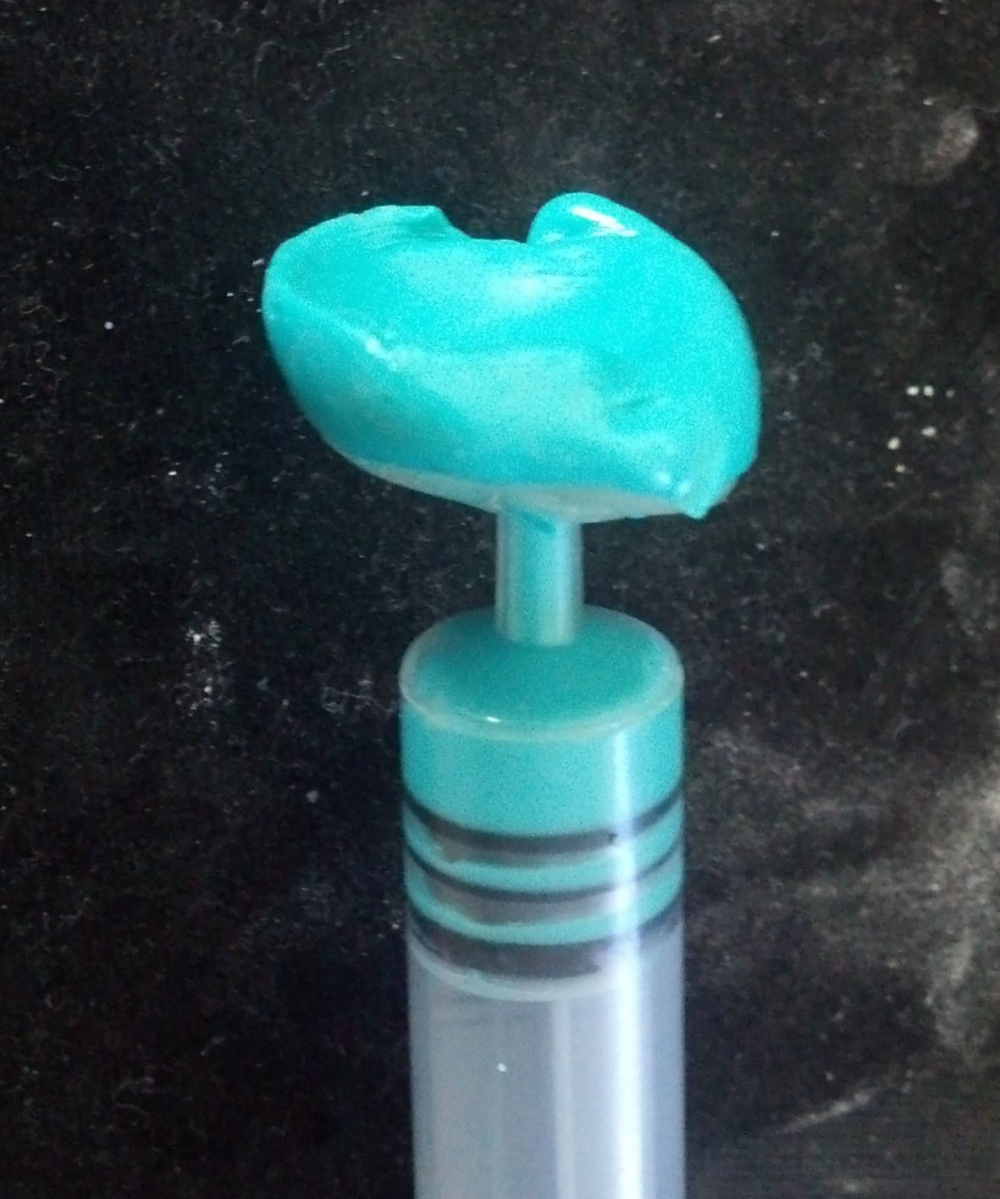
Final impression with additional silicon light body

Additional silicon light body impression material was used as the secondary impression material with the special tray while the patient was gazing straight ahead (Figure 5).

**Figure 6:**
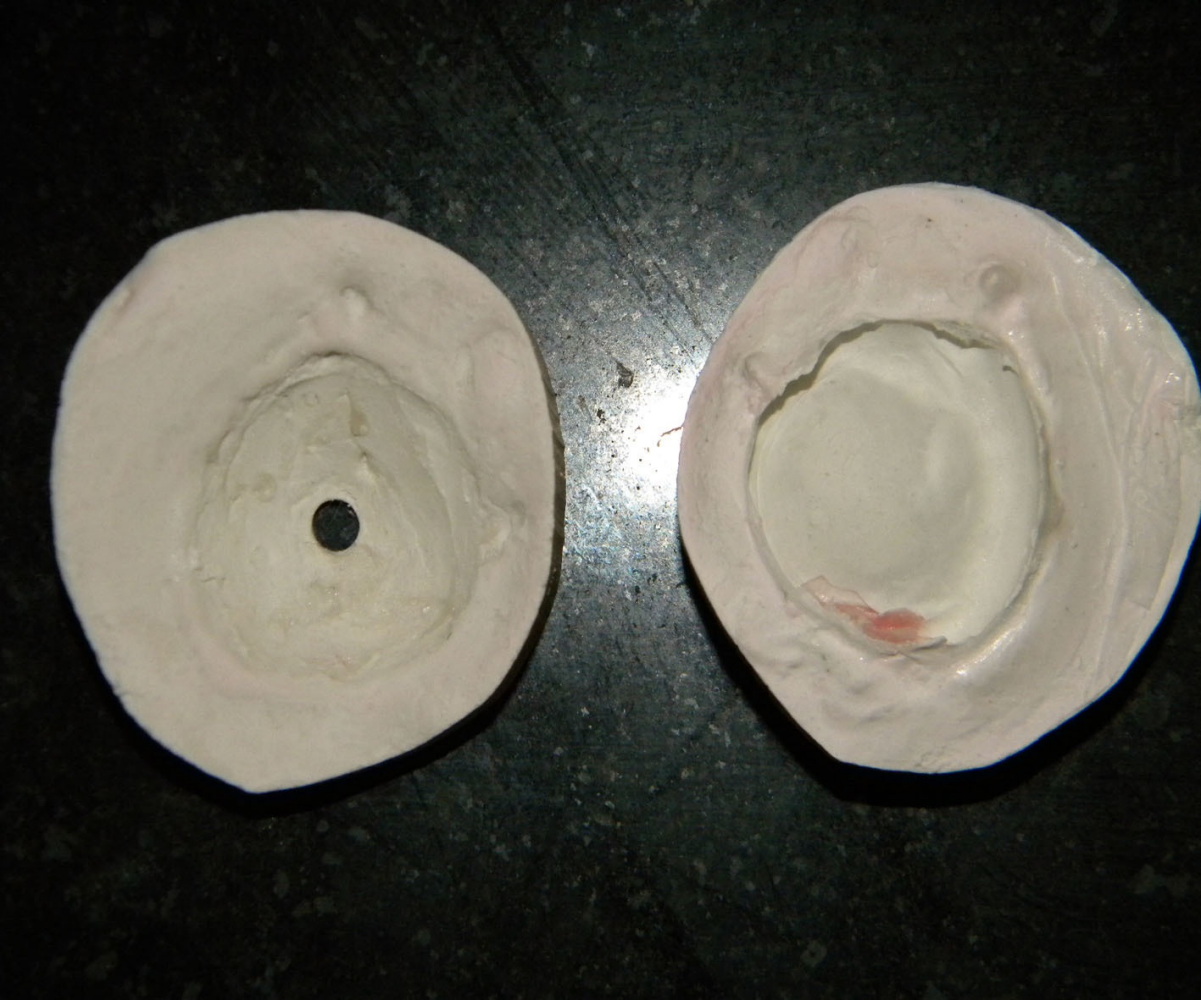
Final cast

The impression was checked for an accurate recording of the posterior wall, the position of the palpebral in relation to the posterior wall, and the greatest extent of the superior and inferior fornices of the palpebrae denoting precise impression. The final cast was poured with a type III gypsum product, Orthokal (Kalabhai Karson Pvt Ltd, Mumbai) (Figure 6).

**Figure 7:**
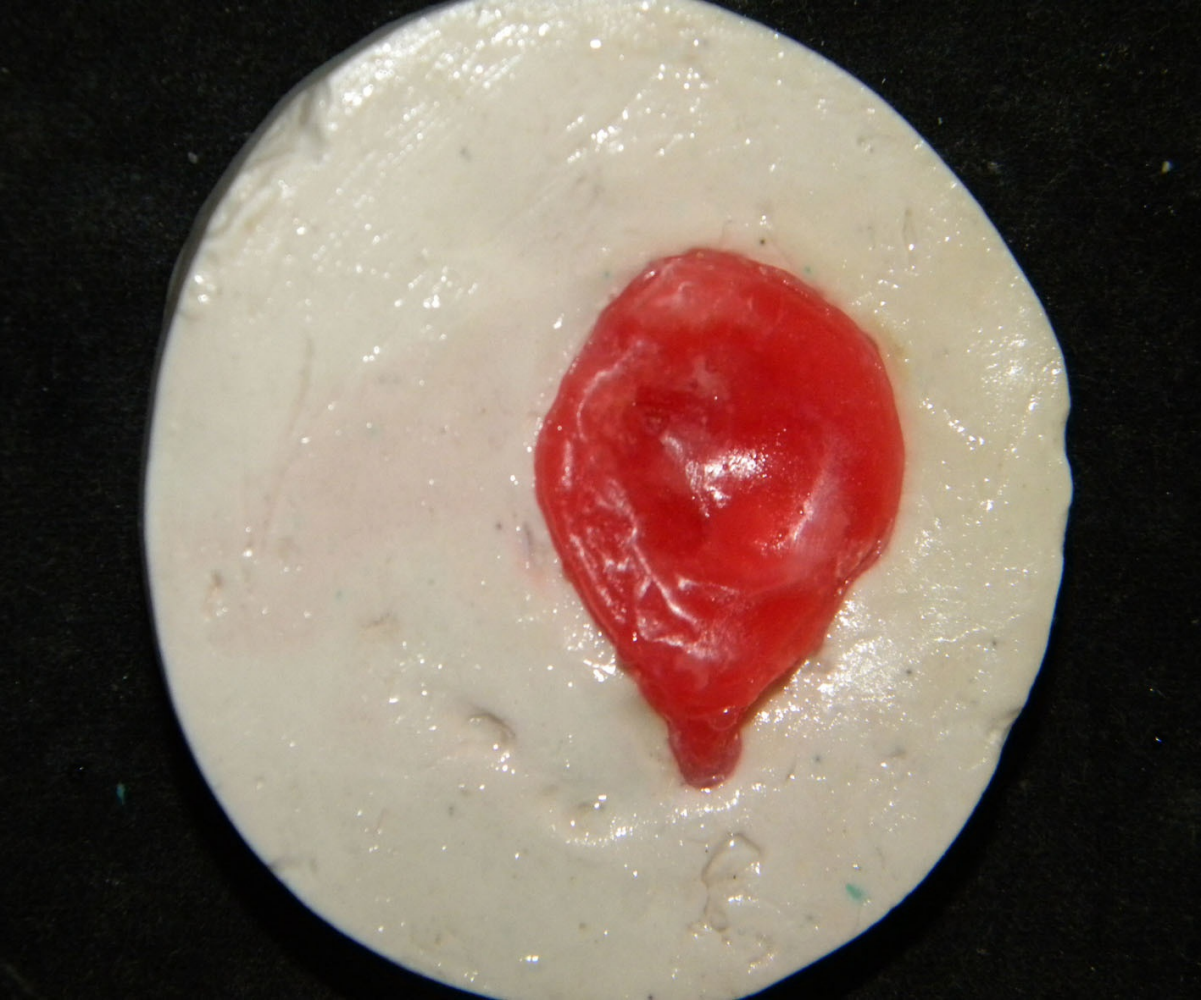
Wax pattern for ocular prosthesis

The sclera was meticulously carved with modeling wax No.2 (Hindustan Dental Products, Hyderabad) to accurately replicate the socket anatomy (Figure 7).

Facial esthetics, comfort, and opening and closing of eyelids were thoroughly evaluated and matched with the companion eye.

**Figure 8:**
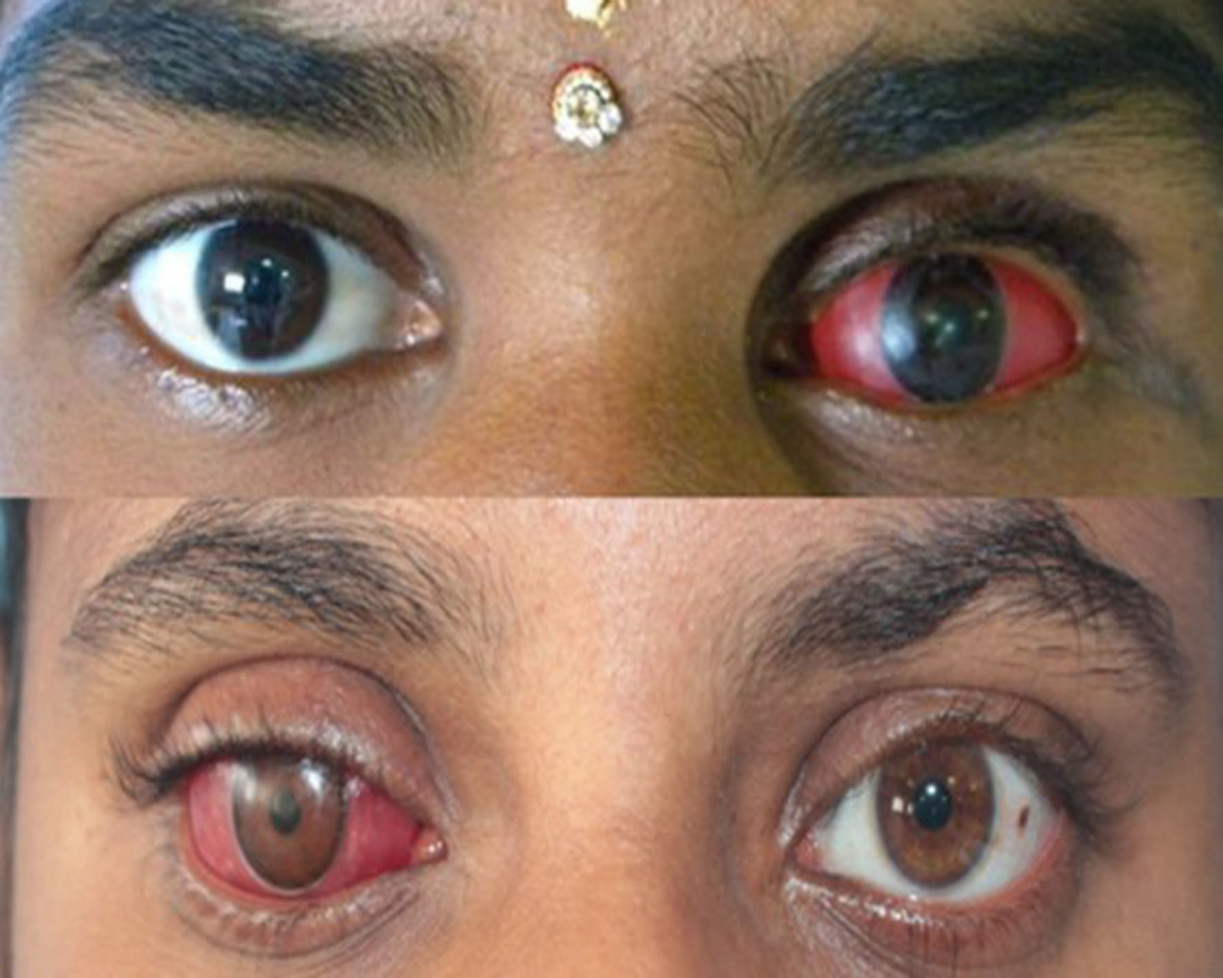
Final wax pattern try-in for Cases 1 and 2 with iris buttons positioned

To mark the iris position, the patient was instructed to gaze straight at an object kept at a distance of four feet [6]. The distance from the iris centre of the natural eye to the facial midline and to the outer canthus was marked and same was reproduced on the defect side. An iris button closely resembling the color, size, and shape of the companion iris was selected and placed in the marked position. Final try-in was done to check the esthetics, iris position, eyelid contours, and opening-closing eye movement (Figure 8).

**Figure 9:**
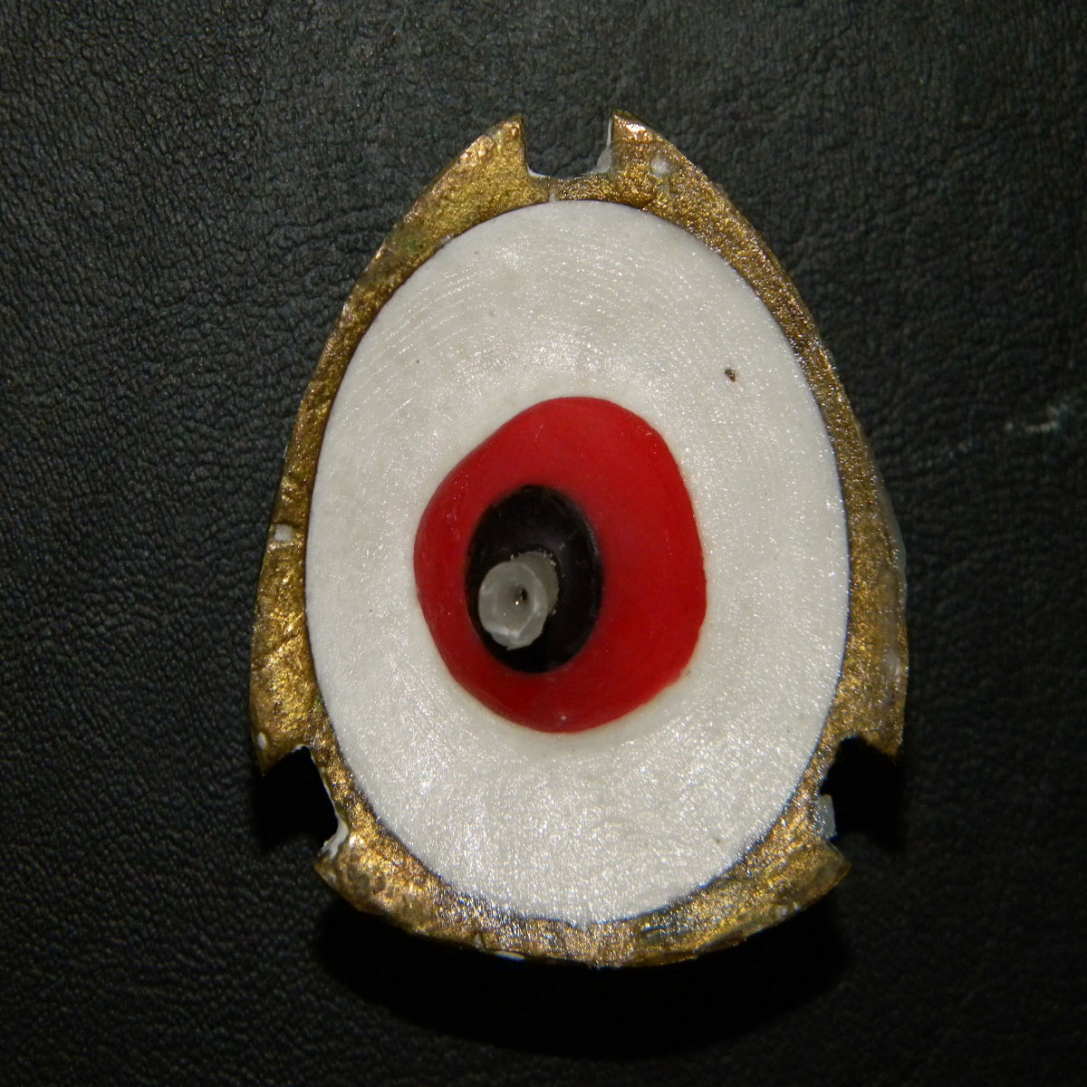
Stabilization of iris before dewaxing procedure

**Figure 10:**
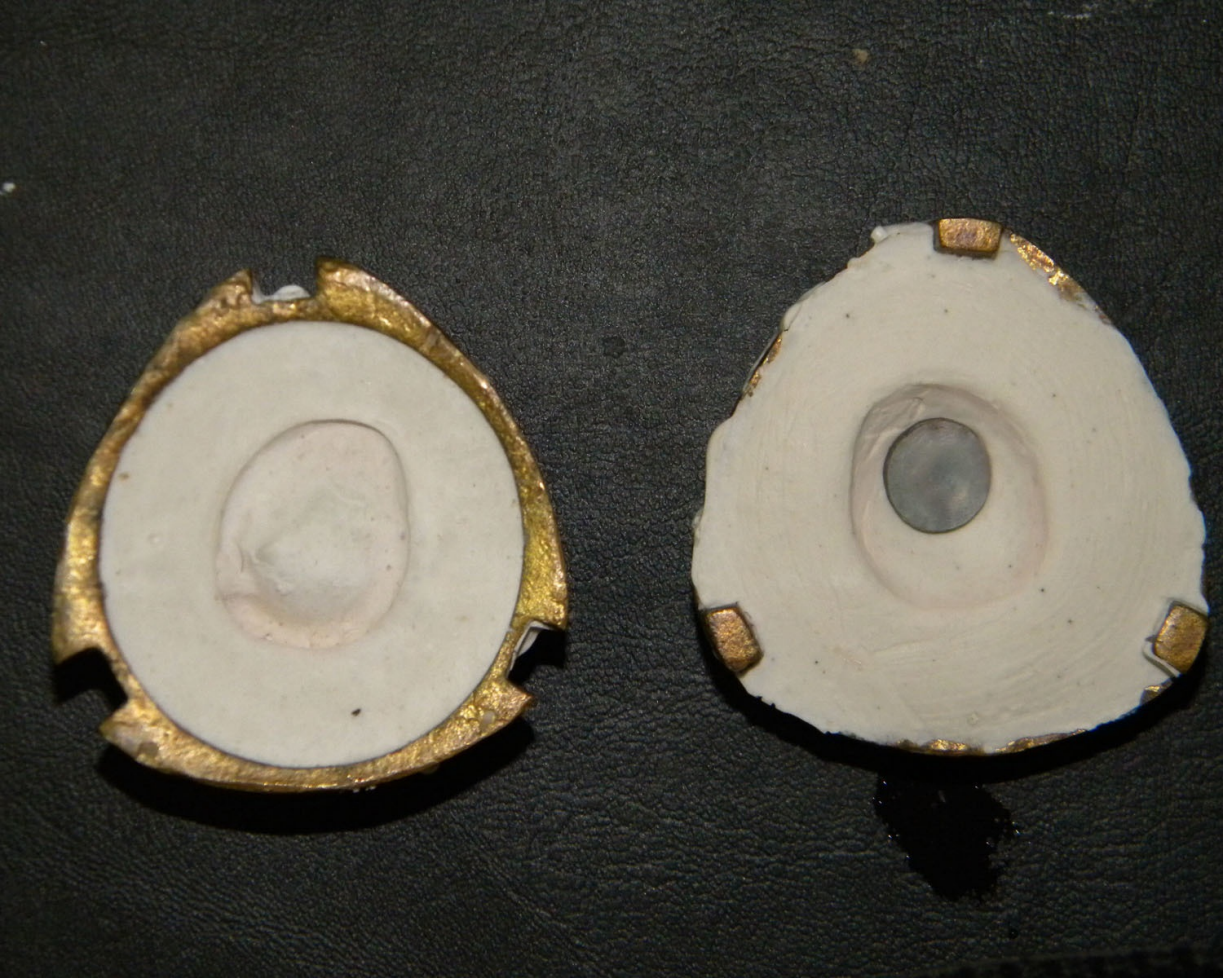
Dewaxed mold

An acrylic resin stem was positioned in the center of the iris button in the wax pattern to stabilize it during de-waxing (Figures 9, 10).

**Figure 11:**
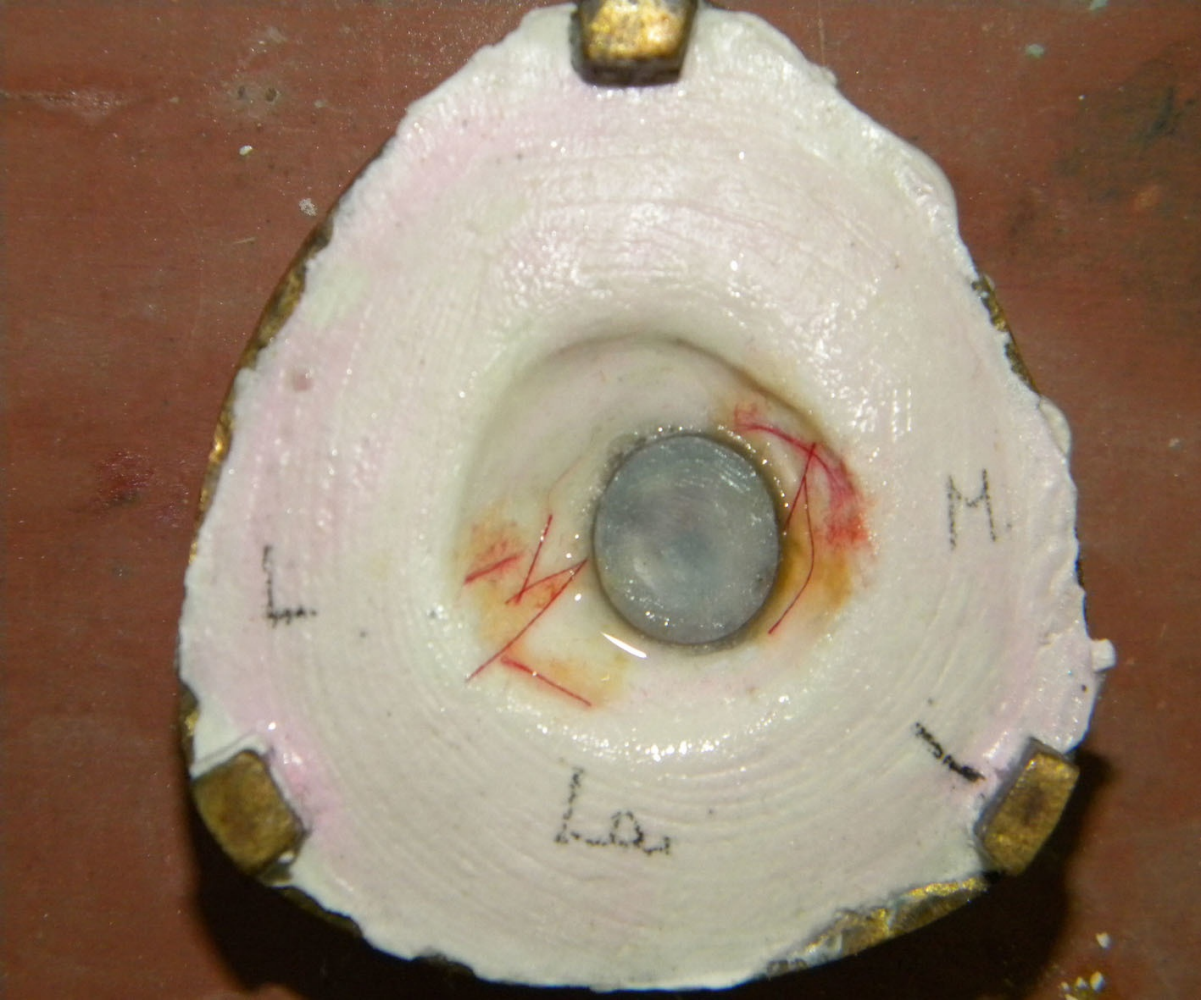
Characterization in the mold

Characterization was done directly on the mold with the patient's presence to compare with the natural eye. A Monopoly syrup prepared with a heat cure monomer to polymer ratio 10:1 was used during characterization to stabilize the acrylic color pigments and the delicate red silk fibers, which were used to replicate the sclera and veins (Figure 11).

**Figure 12:**
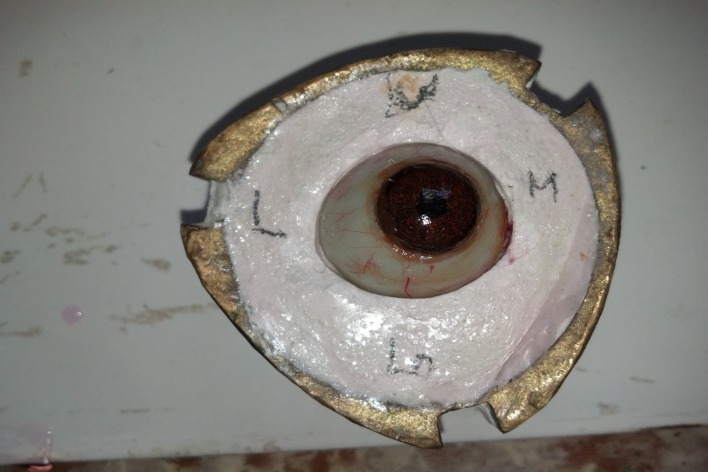
Finishing of the ocular prosthesis

The scleral color was achieved by mixing heat cure clear and tooth-colored acrylic in a 1:1 ratio with color pigments. After processing, the prosthesis was retrieved, trimmed, and highly polished before insertion (Figure 12).

**Figure 13:**
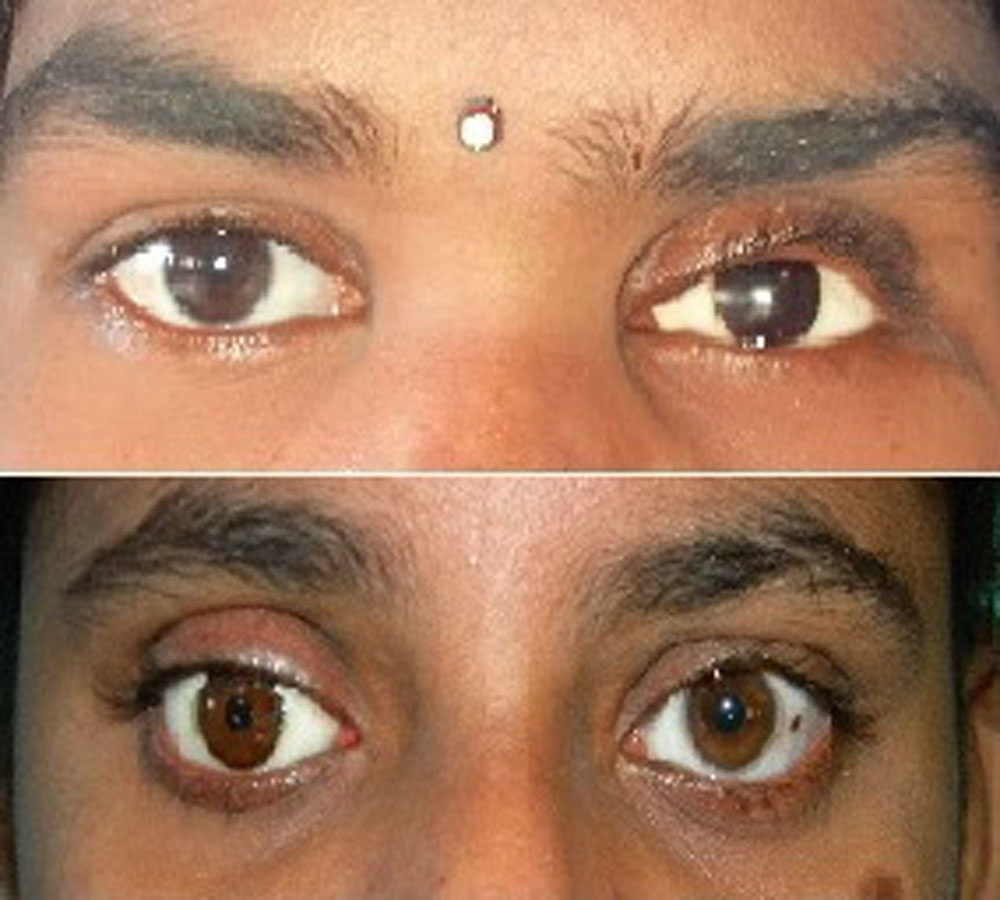
Final ocular prosthesis for Cases 1 and 2

Prosthesis movements were satisfactory and the patient was comfortable (Figure 13).

Post-insertion instructions were given. The patients were reviewed after 24 hours, one week, one month, and six months and no scleral inflammation or pain on eye movements were noted.

## Discussion

Acrylic ocular implants, along with a custom ocular prosthesis, were used to restore the lost orbital volume and help to simulate natural eye movements and esthetics in both our present cases with highly satisfactory results. Studies have shown that an ocular implant provides 65% - 70% of the volume of the lost eye and the remaining volume 30% - 35% being the ocular prosthesis [7]. The nonintegrated acrylic ocular implants covered with donor sclera permitted ﬁxation of the extraocular recti muscles, which improved implant motility but did not allow direct mechanical coupling between the implant and the artiﬁcial eye as discussed in previous studies [8-9]. The efficiency of transmitting movement from the implant to the prosthesis determines the degree of prosthetic motility. The motility may be due to the force between the spherical implants and the prosthesis through the surface tension at the closely fitted conjunctival–prosthetic interface and movements of the fornices as seen in previous studies [9]. The custom ocular prosthesis provided the desired smooth and accurately fitting implant-prosthesis interface, which helped in simulating the fine movements in both cases. An accurate impression is the key to a successful ocular prosthesis. The modified stock tray impression material described by Allen and Webster [10], though it supports the impression material, may not conform properly to the individual's eyeball. The special impression procedure with custom tray helped us achieve intimate contact with the tissue bed without inflammation, scarring, or excessive watering of the eye. The iris is the centre point and of utmost esthetic value in an ocular prosthesis. The preformed iris button helped us achieve good esthetics, eliminated working time, artistic skills, and cost, which occurs in the conventional oil paint and monopoly iris painting technique or digital imaging.

## Conclusions

Custom ocular prosthesis and orbital implants, with the help of special impression techniques, can restore the esthetics and eye movements with better patient compliance.
